# WHO guide on the economic evaluation of influenza vaccination

**DOI:** 10.1111/irv.12510

**Published:** 2017-12-27

**Authors:** Anthony T. Newall, Nathorn Chaiyakunapruk, Philipp Lambach, Raymond C. W. Hutubessy

**Affiliations:** ^1^ School of Public Health and Community Medicine Faculty of Medicine University of New South Wales (UNSW) Sydney Australia; ^2^ School of Pharmacy Monash University Malaysia Selangor Malaysia; ^3^ Center of Pharmaceutical Outcomes Research (CPOR) Department of Pharmacy Practice Faculty of Pharmaceutical Sciences Naresuan University Phitsanulok Thailand; ^4^ Asian Centre for Evidence Synthesis in Population Implementation and Clinical Outcomes (PICO) Health and Well‐being Cluster Global Asia in the 21st Century (GA21) Platform Monash University Malaysia Bandar Sunway Selangor Malaysia; ^5^ Initiative for Vaccine Research World Health Organization Geneva Switzerland

**Keywords:** guidelines, influenza vaccines, vaccine economics

## Abstract

Influenza is responsible for substantial morbidity and mortality across the globe, with a large share of the total disease burden occurring in low‐ and middle‐income countries (LMICs). There have been relatively few economic evaluations assessing the value of seasonal influenza vaccination in LMICs. The purpose of this guide is to outline the key theoretical concepts and best practice in methodologies and to provide guidance on the economic evaluation of influenza vaccination in LMICs. It outlines many of the influenza vaccine‐specific challenges and should help to provide a framework for future evaluations in the area to build upon.

## 1 INTRODUCTION

Influenza is responsible for substantial morbidity and mortality across the globe, with a large share of the total disease burden occurring in low‐ and middle‐income countries (LMICs).^1^ The cost‐effectiveness of seasonal influenza vaccination programmes has been widely assessed in high‐income countries.^2^, ^3^ The value for money estimated for programmes targeted at children,^4^ the elderly^5^ and those at high risk of infection and/or severe complications^6^ has been most favourable, whereas results for healthy adults have been less consistent.^7^

A recent literature review on the topic found that in LMICs relatively few economic evaluations have assessed the value of seasonal influenza vaccination.^8^ Nine economic evaluations were identified in middle‐income countries, with none identified from low‐income countries.^8^ The review found important methodological limitations in several studies and called for greater standardization of methods for economic evaluation of influenza vaccination, and thus for the need of global guidance on the economic evaluation of influenza vaccination in LMICs.

In response, WHO commissioned the *Guidance on the economic evaluation of influenza vaccination*,^9^ which outlines the key theoretical concepts and best methodological practice, aiming to offer high‐level guidance on influenza vaccination assessment in LMICs (see Box 1). The guide is aligned to existing vaccine introduction guidance^10^ and key documents for the assessment of influenza vaccination (Table 1).

Box 1 **Summary of methodological recommendations from guidelines**

**Disease burden**

- Ideally, at least 5 years of data should be used to estimate the existing influenza disease burden.^12^ However, a shorter period (minimum of a single calendar year) can serve as a starting point.
- WHO's *A manual for estimating disease burden associated with seasonal influenza* 12 can be used to estimate some key outcomes, but further sources are required (eg, to estimate non‐medically attended influenza).

**Economic burden**

- Evaluations should ideally adopt a societal perspective, including all relevant costs and consequences irrespective of who incurs them. However, costs borne by different entities should be reported separately where possible.^17^
- If productivity costs are included, they should be reported separately from other costs and cost‐effectiveness results should be presented with and without indirect costs.

**Programme costs**

- The vaccine administration strategy should be carefully considered and outlined in detail. Key choices include who administers the vaccine, in what setting and whether it is delivered opportunistically or at a separate encounter.
- Where possible, estimates of adverse events following immunization (AEFI) should be included in economic evaluations of influenza vaccination.

**Programme impact**

- Efficacy against confirmed influenza disease from a meta‐analysis will often be the most appropriate estimate. This can be applied to all estimates of influenza‐specific outcomes (eg, influenza death).

**Modelling approach**

- Table 2 summarizes when to consider each modelling approach.

**Discounting/horizon**

- Costs and effects should be discounted at the appropriate level indicated in relevant guidelines for the setting under evaluation, but results should also be reported applying WHO‐CHOICE recommendations in sensitivity analysis.
- A 1‐year time horizon may be appropriate in most cases; however, long‐term consequences from prevented influenza mortality that occur outside the 1‐year time frame must be fully incorporated into model results.

**Results/presentation**

- In most cases, strategies for each different target group (eg, pregnant women) should be compared only to alternative strategies for that group.
- Total costs and outcomes should be presented for each strategy, as well as the incremental results comparing the strategies. These results should be further disaggregated to show the factors driving the results.

**Uncertainty**

- Key types of uncertainty, including parameter, methodological and structural, should be explored. Interyear variation also needs to be considered.^33^

**Other recommendations**

- Consideration should be given to specific issues that may arise when evaluating a particular population subgroup (see^2^ for a summary of potential issues).

**Table 1:** WHO documents and tools that may be relevant to the different subsections of an economic evaluation of influenza vaccination

Category	Publication	What it provides
Burden of disease	A manual for estimating disease burden associated with seasonal influenza	A standardized tool to estimate the respiratory burden of influenza
Economic burden	Manual for estimating the economic burden of seasonal influenza	A step‐by‐step guide and costing tool to estimate the cost of influenza
Programme cost	Maternal seasonal influenza vaccination programme planning and costing tool	Specific steps and tools to cost maternal influenza vaccination delivery programmes
	Guidelines for estimating costs of introducing new vaccines into the national immunization system	A stepwise approach to estimating incremental vaccination programme costs
	WHO‐UNICEF guidelines for developing a comprehensive multiyear plan (cMYP)	Steps to develop a cMYP including planning and costing tools
Economic evaluation	Guidance on the economic evaluation of influenza vaccination (*current document*)	Specific guidance for the economic evaluation of influenza vaccination
	Guide for standardization of economic evaluations of immunization programmes	General guidance on the economic evaluation of vaccination programmes
Strategic health planning	WHO OneHealth Tool	Supporting sector‐wide integrated strategic health planning, costing and health impact analysis

## 2 ESTIMATING THE DISEASE BURDEN AND ASSOCIATED HEALTHCARE USE

Estimating the disease burden from influenza using routinely collected data can be challenging. This is the case even in high‐income countries with comprehensive surveillance networks and national electronic healthcare records (eg, for hospitalization episodes). One major reason for this is that laboratory confirmation is not routinely requested in suspected influenza cases. Estimation is further complicated because patients may present with secondary complications potentially triggered by influenza (eg, acute myocardial infarction).^11^

**Figure 1:**
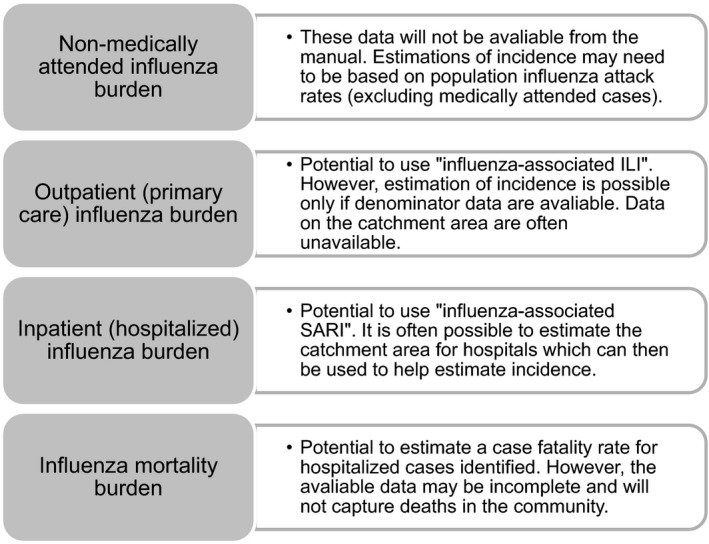
Elements of the influenza disease burden that may/may not be estimated using WHO's *A manual for estimating disease burden associated with seasonal influenza* 12

WHO's *Manual for estimating disease burden associated with seasonal influenza* 12 outlines various methods that can be applied in LMICs to evaluate the disease burden attributable to influenza. However, other sources of data will be required to estimate the full range of influenza disease burden (see Figure 1). Using the definitions set out in this manual, the estimated disease burden is divided into 2 main categories: (i) influenza‐associated ILI, which represents an estimate of the outpatient/primary care clinic visits due to influenza illness, and (ii) influenza‐associated severe acute respiratory infections (SARI), which represents an estimate of the hospitalization visits due to influenza illness. Both categories use laboratory confirmation (on at least a subset of cases) to estimate the proportion of events suspected to be due to influenza. Evaluating mortality in SARI cases can also be used to estimate the case fatality rate in hospitalized influenza‐positive cases.^12^ However, such an estimate is likely to be a conservative as some deaths from influenza infection will not occur in a hospital setting.

An alternative approach is to use statistical modelling techniques to estimate the influenza‐attributable burden.^13^, ^14^, ^15^ These methods involve time series analyses of non‐specific disease outcomes, such as respiratory deaths, to estimate a non‐influenza baseline burden above which any excess disease may be considered attributable to influenza. While these methods are a useful way to estimate influenza‐attributable burden, they have specific data requirements (eg, complete and accurate data on the non‐specific disease outcomes), can involve relatively complex technical analysis and may be more difficult to apply in (sub)tropical regions where influenza does not always show a clear seasonal pattern of circulation.

Year‐to‐year variation in the influenza disease burden should be considered in the analysis. This variation is due to changes in the circulating virus over time which can impact on influenza virus transmissibility and virulence.^4^ It is suggested that data from at least 5 years are used to estimate the existing influenza disease burden^12^; where not available, a minimum of a single calendar year can serve as a starting point provided that appropriate caution is taken when interpreting the results.^12^ In all cases, but particularly when dealing with imperfect data, care should be taken to conduct an appropriate sensitivity analysis across a range of plausible values (see Section 8). Age must also be considered when estimating influenza disease burden, with age‐specific rates used whenever possible.

Non‐medically attended influenza cases have been found to be influential in many economic evaluations of influenza vaccination in high‐income settings.^2^ While costs and consequences attached to each non‐medically attended case may be relatively small, the large number of cases can mean that they have an important impact on cost‐effectiveness. In LMICs, some individuals within the society may face cost and health access issues and may either seek no care, self‐medicate or seek informal care. However, these cases may still result in indirect (productivity) costs and/or other direct healthcare costs (eg, out‐of‐pocket medication costs).^16^

Unless specific estimates already exist for the target population, options to estimate the non‐medically attended influenza burden are limited without commissioning potentially expensive empirical data collection or complex modelling studies. Nevertheless, plausible estimates should be included, at least in sensitivity analysis, to allow for an understanding of their impact on evaluation results. Estimates of the total symptomatic attack rate can be used to calculate the non‐medically attended symptomatic disease rate by excluding estimates of medically attended cases.^12^

It is generally recommended that a cost‐utility approach be used for economic evaluations.^17^ All of the cost‐utility analyses identified in LMICs to date have used a quality‐adjusted life years (QALYs) framework.^8^ However, in some cases, disability‐adjusted life years (DALYs) may be a more appropriate outcome measure than QALYs for LMICs as estimates may be more consistently available across all countries.^17^ Local guidelines and the availability and/or transferability of quality‐of‐life weights to the setting under evaluation should help inform these decisions. Careful thought must also be given to whether DALYs or QALYs are the more appropriate measure to value (uncomplicated) acute influenza illness.

## 3 ESTIMATING THE ECONOMIC BURDEN ASSOCIATED WITH INFECTION

Detailed advice on how to calculate the cost of influenza disease can be found in the WHO *Manual for estimating economic burden of seasonal influenza*.^18^ This manual offers a step‐by‐step guide to help analysts estimate the economic burden of influenza in their setting. It separates influenza‐associated costs into direct medical, direct non‐medical and indirect costs. The unit costs calculated as part of this manual may useful in economic evaluations of influenza vaccination. However, estimates have to be appropriate for the group(s) that is being evaluated.

The *WHO Guide for standardization of economic evaluations of immunization programmes* 17, 19 recommends that evaluations adopt a societal perspective, including all relevant costs and consequences irrespective of who incurs them. However, costs borne by different entities (eg, local governments, donors) should be reported separately (where possible) within economic evaluations to account for different viewpoints that decision‐makers or audiences may have.^17^

Indirect productivity costs, due to time off work from influenza illness or while caring for those ill (eg, children), have been found to be influential in high‐income settings.^2^, ^4^, ^6^, ^7^ In LMICs, the costs of a day off work may be substantially lower compared to high‐income countries but should still be considered if they fit within the perspective adopted. If included, indirect costs should be reported separately and cost‐effectiveness results be presented with and without their inclusion.^17^

## 4 ESTIMATING THE COSTS ASSOCIATED WITH THE VACCINATION PROGRAMME

Influenza vaccination may incur different costs depending on the type of vaccine used (live‐attenuated vaccines [LAIV], trivalent inactivated vaccines [TIV] or quadrivalent inactivated vaccine [QIV]). The service delivery approach can also impact on the cost‐effectiveness of influenza vaccination strategies.^8^ Due to the cost of physician's visit/time, administration in a medical setting (eg, healthcare facility) can involve higher incremental costs compared to non‐medical administration sites (retail [pharmacy] sites, workplace vaccination or school‐based vaccination programmes). However, costs may be reduced if vaccination can be administered opportunistically or by lower wage staff (such as nurses or other qualified staff).

The WHO Strategic Advisory Group of Experts (SAGE) on Immunization identified risk groups for influenza, including those at increased risk of exposure to influenza virus (healthcare workers) and those at particular risk of developing severe disease (“pregnant women, children aged <5 years, the elderly and individuals with underlying health conditions such as HIV/AIDS, asthma and chronic heart or lung diseases”).^20^ Pregnant women, children aged 6 months to 2 years and healthcare workers are likely to be present in the health system already for unrelated reasons and could receive influenza vaccine in an opportunistic manner at a relatively low incremental administration cost.^20^, ^21^ However, additional complexities such as the seasonal timing of influenza vaccination and the potential requirement for 2 doses in eligible unvaccinated children^21^ must be considered.

The level of uptake (coverage) for influenza programmes can be hard to predict in advance and may be affected by the delivery approach and target group. Unlike adding a new vaccine to an infant schedule, where estimates of the uptake of existing vaccines in the setting may provide a template to predict demand, current influenza vaccines require annual revaccination and may target previously unvaccinated groups. Hence, vaccine uptake may be lower than rates achieved in traditional infant schedule vaccines. Risk and benefit perceptions about influenza disease and vaccination in both vaccinee and vaccinators may also impact on uptake.

To increase uptake of vaccine among different target groups, additional efforts will be needed in terms of information, education and communication and social mobilization. Outreach vaccination strategies may also play an important role in some regions to vaccinate remote or hard‐to‐reach populations. Estimating cost consequences of these activities will be crucial in economic evaluations of influenza vaccination. Due to challenges identifying target populations, for example patients' chronic conditions, uptake of vaccination strategies targeting specific risk groups in high‐income countries has often been relatively low^22^ and can be expected to be even lower in LMICs. Strategies targeted to obtain a high uptake in such groups should include additional costs for screening those suitable for vaccination.

Patient indirect productivity costs and direct (non‐medical) transportation costs may also be considered when evaluating the cost of a vaccination strategy. Including productivity costs, in terms of lost time to patients or caregivers to allow receipt of influenza vaccination, will increase the costs attributable to the programme. Including transportation costs paid by individuals to attend vaccination will also add to the total societal costs of the influenza vaccination strategy. However, in some cases, the incremental cost to individuals may be insignificant and may not warrant inclusion (eg, for opportunistic influenza vaccination at an existing healthcare visit).

Severe adverse events following immunization (AEFI) for influenza vaccination are very rare and mild adverse events usually resolve after a short period of time.^23^, ^24^, ^25^, ^26^ Due to the relatively minor consequences from these events, economic evaluations have not always included AEFI and, if included, they have generally not been found to be influential in determining cost‐effectiveness.^4^ Nevertheless, estimates of AEFI should be included in economic evaluations of influenza vaccination where possible, in particular if vulnerable populations such as pregnant women,^27^, ^28^ or younger children or those with certain underlying conditions,^21^, ^26^ are targeted.

## 5 ASSESSING THE IMPACT OF VACCINATION EFFORTS

Influenza vaccine efficacy estimates should generally be obtained from randomized clinical trial evidence, ideally from meta‐analyses that appropriately synthesize all the relevant available data rather than from a single vaccine trial.^2^ Estimates of efficacy should incorporate multiple influenza seasons because of the year‐to‐year variation in vaccine match as well as in transmissibility and prior immunity in the population.^4^ Efficacy estimates may differ depending on the population group being targeted, for example by age and for those with underlying medical conditions.^21^, ^22^, ^23^, ^24^ Distinctions should also be made between the different types of influenza vaccines (eg, between LAIV and TIV, and adjuvanted and quadrivalent vaccines). There may also be differences in vaccine efficacy between regions.^29^

Efficacy estimates against non‐specific outcomes have sometimes been used in economic evaluations of influenza vaccination programmes,^2^, ^4^ but the most straightforward estimate is efficacy against confirmed influenza disease. It is reasonable to apply a vaccine efficacy calculated against confirmed influenza disease to all estimates of influenza‐specific outcomes, including influenza hospitalizations and influenza deaths. This involves simplifying assumptions, for example, that the prevention of influenza infection will prevent all subsequent disease outcomes from this infection. Efficacy estimates specifically against severe influenza outcomes cannot easily be measured in clinical trials because of the large sample sizes required to detect an adequate number of events.^30^

The probability of vaccine match to the circulating strains must be accounted for when evaluating the cost‐effectiveness of influenza vaccination strategies. While the vaccine may match in any given year, over the longer term the match to the predominate strains will not always be successful.^31^ The vaccine match is important as it has been shown that a poor match will reduce the efficacy of the vaccine to prevent influenza illness.^23^, ^24^, ^25^ Economic evaluations can apply a vaccine efficacy estimate from a meta‐analysis that already incorporates both matched and poorly matched seasons (eg, they may apply a single estimate of vaccine efficacy derived from trials run over multiple years). In many cases, this method may be a reasonable approach; however, it can be problematic when using modelling techniques that seek to estimate herd protection effects.^32^, ^33^

An additional complexity is introduced when evaluating the impact of influenza vaccination where there is no clear seasonal influenza activity. Here, a significant proportion of the annual influenza burden may occur before influenza vaccine can be administered. In (sub)tropical regions, economic evaluations therefore have to take seasonal variability into account and make appropriate model assumptions to ensure realistic predictions of vaccination impact.

Influenza immunization programmes have the capacity to help protect the population also through reduced transmission in the community and within households.^34^, ^35^ Herd protection effect estimates can be included in economic evaluations of influenza vaccination through the use of dynamic transmission models (see Section 6). However, constructing these models can be complex and sometimes a proxy form of herd protection has been included in influenza economic evaluation models.^4^ This approach may involve the application of a “static” (fixed) reduction in disease, based on empirical trial evidence to contacts of vaccinated persons. For instance, an indirect protection effect might be applied to household contacts of vaccinated children. However, this method may be potentially misleading if the estimate of indirect protection is being transferred to a setting which differs in important ways from the trial.^4^ If proxy estimates are used, uncertainty in this estimate should be considered, and it may be more appropriate to apply estimates of proxy herd protection only in sensitivity or scenario analysis (Section 8).

Another important factor that impacts population protection from influenza vaccination is the vaccine uptake in the targeted groups. The degree of uptake impacts the total direct protection and may also (depending on those targeted) have a substantial impact on any indirect protection of the community through herd protection. In models not incorporating herd protection, uptake will have an impact on the absolute benefits of vaccination and the total cost of the programme but the impact on the cost‐effectiveness ratio may not always be as substantial. This is because the costs of the vaccination programme and the health benefits that accrue through the programme may both increase (approximately) proportionally with the uptake.^17^ However, with substantial fixed programme costs or economies of scale (eg, when purchasing large orders), obtaining higher coverage may improve the cost‐effectiveness of the programme. When herd protection is modelled, vaccination uptake can become more influential in determining cost‐effectiveness^36^ (see Section 6).

## 6 ALTERNATIVE MODELLING APPROACHES

The simplest approach to evaluate the cost‐effectiveness of influenza vaccination is to apply a “decision tree” model (Table 2). In these models, each pathway through the “tree” represents a sequence of events and is associated with costs and consequences.^37^ Decision trees are often used when the costs and consequences of an intervention occur over a short period of time, as is the case for influenza vaccination. This is because decision tree models cannot explicitly account for time. However, as is discussed in Section 7, this may not be essential in influenza models as the impact of long‐term consequences from mortality can be incorporated through a discounted pay‐off attached to specific endpoints where required.

**Table 2:** Alternative approaches to the economic evaluation of influenza vaccination

Assessment approach	Advantages for influenza vaccination evaluation	Disadvantages for influenza vaccination evaluation	When to consider using this approach
Static decision tree model	Adequate to assess most influenza strategies Relatively simple to construct and interpret	Unable to predict herd protection effects Unable to explicitly incorporate time	Vaccinated groups unlikely to change population disease transmission substantially Dynamic modelling is impractical due to cost, etc.
Static Markov model	Relatively simple to construct and interpret Allows the explicit inclusion of time	Unable to predict herd protection effects	See above Need to model time explicitly (eg, when dose number varies by previous year's vaccine status)
Dynamic transmission model	Able to predict herd protection effects	Increased complexity to build and interpret results Time‐consuming and more costly to construct Has additional data requirements	Vaccinated groups likely to change population disease transmission substantially (eg, children) Expertise, time and data are available to facilitate dynamic modelling
Economic evaluation alongside clinical trial	Can facilitate collection of resource use and quality‐of‐life data	Unable to predict herd protection (non‐cluster trials) Unlikely to capture rare events (eg, influenza death) May not capture interyear variability from influenza	An economic evaluation can be added (“piggyback”) on to a clinical trial already planned in the setting Existing data from a clinical trial can be used

In most circumstances, “Markov” state‐transition models with a static (fixed) force of infection irrespective of the proportion of the population that is infectious have limited advantages over decision tree models in the context of influenza evaluations. This type of state‐transition model allows for time‐ or age‐dependent transition probabilities to be specified and is therefore often appropriate when costs and consequences occur over an extended period (eg, as in chronic diseases).^37^ However, the duration of influenza vaccine protection is typically modelled as lasting only for a single season because of strain changes that occur from season to season. There are some situations where this type of model may be advantageous; for example, it can be used to explore alternative options for the timing of vaccination, where vaccination uptake can be modelled as a gradual process.

Dynamic transmission models can incorporate herd protection into economic evaluations by having the risk (force) of infection vary (being dynamic rather than static) on the basis of the proportion of the population that is infectious over time.^38^, ^39^ These models are increasingly used in economic evaluations of influenza vaccination^4^; for example, recently a dynamic model was used to assess the cost‐effectiveness of influenza vaccination of children in Thailand.^40^ However, dynamic models are often more complex, time‐consuming and costly to produce than static models and have additional data requirements (eg, information on contact patterns between individuals).^32^ As such, these models may not be the most appropriate choice for evaluations in LMICs in all circumstances.

Dynamic transmission models are most applicable when evaluating programmes targeting a substantial proportion of those responsible for influenza transmission (eg, vaccinating all eligible children^34^) and are less likely to be required when evaluating programmes that are less likely to result in substantial herd effects (eg, when targeted on relatively small population subgroups). In most cases, the use of static models will bias an analysis towards conservative estimates of cost‐effectiveness.^17^ The *WHO guide for standardization of economic evaluations of immunization programmes* provides an informative decision chart to identify what type of model may be appropriate in different circumstances.^17^ This guide also provides important information on model validation and collaboration.^17^

Economic evaluations alongside clinical trials provide another avenue to assess the cost‐effectiveness of influenza vaccination strategies.^8^ However, there may be several important limitations to this approach^2^, ^4^ (see Table 2).

## 7 DISCOUNTING AND ANALYTICAL HORIZON

The majority of the costs and consequences resulting from influenza vaccination occur within a single year, making discounting less influential compared to vaccination programmes with a longer delay between upfront costs of the vaccination programme and the benefits derived from prevented illness. In influenza evaluations, the impact of discounting is often most important to account for the long‐term consequences that accrue from prevented influenza mortality. While the prevention of deaths occurs in the year of vaccination, the life years (or DALYs/QALYs) and/or any productivity gains included from prevented mortality accrue over time and should be discounted at the appropriate level indicated in relevant guidelines for the setting under evaluation. To be consistent with current WHO‐CHOICE recommendations, in sensitivity analysis (Section 8), a discount rate of 3% for both costs and effects (alternative 0%) should also be applied.

The analytical horizon for economic evaluations should be long enough to account for differences in costs and consequences between the various strategies being evaluated^37^ (eg, between “no influenza vaccination in group X” and “influenza vaccination targeted at group X”). As the majority of costs and consequences resulting from influenza vaccination occur in a single year, a 1‐year time horizon may be appropriate for the economic evaluation. However, it is important that the long‐term consequences from prevented influenza mortality that occur outside of this single‐year time frame are fully incorporated into model results. One simple way to do this is to apply discounted pay‐off/s in the model that incorporate the full benefits of prevented influenza mortality. It should be noted that longer time horizons are often required in more complex modelling approaches (Section 6), such as those which follow populations through time to account for the build‐up of immunity and herd protection.

## 8 ESTIMATING AND PRESENTING RESULTS OF THE ECONOMIC EVALUATION

The influenza strategy being considered for implementation should be compared to an appropriate alternative. For example, the alternative for comparison may be the costs and consequences of “no vaccination” (i.e do nothing) for this group. This will allow an incremental cost‐effectiveness ratio (ICER) to be calculated, representing the difference in costs between the alternatives divided by the difference in health outcomes.^37^ However, there may be more than 2 strategies that should be considered for this target group; for instance, one may also want to consider immunization with an alternate vaccine (eg, LAIV or TIV) in this group. The ICERs should then be calculated by comparing each strategy to the next best alternative, after excluding dominated strategies (see^17^ for detailed advice on this process). Only strategies that are mutually exclusive would generally be considered as comparators within a single economic evaluation.^37^

In most situations in LMICs, a practical approach to economic evaluation is to treat each target group as independent (eg, pregnant women, children aged 6 months to 2 years, children aged 2‐5 years). In this way, a different economic evaluation would be completed for each group that may be considered for vaccination, and within each evaluation, the influenza vaccination strategy would be compared only to alternatives for that group. The ICER results for each evaluation can then be interpreted separately against an appropriate threshold. If applying a dynamic modelling framework, there is also the potential to evaluate influenza strategies for different target groups against each other, accounting for herd effects that the vaccination of one group can have on another and on the wider population.^3^

Costs and outcomes should be presented in a detailed manner for each strategy being evaluated.^8^ This may include a table presenting the total costs, total outcomes (eg, QALYs) for each strategy, as well as incremental results comparing the strategies under consideration. The results should be further disaggregated to allow readers to understand the relative contribution of different factors to these overall results. By breaking down the total costs into different categories of resource use, the different elements contributing to the overall results can be more easily understood (eg, comparing the total cost savings from the prevention of influenza inpatient visits vs the savings from prevention of outpatient visits). The outcomes results should also be disaggregated when they are reported. For example, the total QALYs gained for prevented influenza mortality and the QALYs gained from prevented influenza morbidity could be reported separately.

There is currently no consensus as to what approach should be used to establish thresholds in LMICs. Ideally, thresholds in LMICs should be informed by the alternative ways the health resources could be allocated, as well as local budget constraints.^41^ As a result, it is important to consider the findings of budget impact analyses for the various influenza vaccination strategies under consideration. There may also be locally established thresholds for decision‐makers which should be considered (as in Thailand^42^). The estimation of thresholds for LMICs is an area of research that is evolving quickly, and new recommendations may soon emerge. WHO recommends against imposing a strict threshold as a decision rule for policy options. While cost‐effectiveness ratios are undoubtedly informative in assessing value for money, countries should be encouraged to develop a context‐specific process for decision‐making that is supported by legislation, has stakeholder buy‐in and is transparent, consistent and fair.

## 9 ASSESSMENT OF UNCERTAINTY AND INTERYEAR VARIABILITY

It is vital to assess and appropriately present uncertainty when estimating the cost‐effectiveness of influenza vaccination programmes. Uncertainty in influenza models can be placed into 3 main categories: parameter, methodological and structural (see^43^ for a detailed discussion of these categories). Parameter uncertainty is the most frequently discussed form of uncertainty.^43^ This type of uncertainty reflects doubt about the true (numerical) value of parameter inputs used in an economic model. Parameter uncertainty may often go beyond this sampling uncertainty to include other factors such as the representativeness of the sample. Methodological uncertainty refers to uncertainty in the choices made by an analyst when conducting an economic evaluation.^43^ Structural uncertainty refers to the design of the model and the extent to which it captures the relevant disease and intervention characteristics.^43^ One key form of structural uncertainty for influenza evaluations is the choice of model. While it may not be feasible to explore different model types, if a dynamic model is used the results can be presented both with and without herd protection.

Alongside uncertainty regarding the true average (numerical) value of parameters in influenza vaccination models, there is also variation between influenza seasons in many values. This variation results from the interyear variation in influenza virus transmissibility, virulence, prior immunity and vaccine match.^4^ However, as economic evaluations generally seek to make decisions on whether to implement vaccination programmes for several years, one simple approach is to use of the average input values calculated from data collected over several years as the base‐case value (eg, for hospitalization rates). The most appropriate approach to use should be carefully considered and decision‐makers should be aware that cost‐effectiveness in any given year may vary substantially.

## 10 CONCLUSIONS

Influenza vaccination strategies in LMICs offer substantial scope to reduce both morbidity and mortality. However, there are currently few published economic evaluations for LMICs that can help decision‐makers understand the value for money that may be offered by different influenza vaccination strategies.^8^ As many economic evaluations have been conducted in high‐income countries,^2^ some of the lessons learned through this process can help to inform future evaluations for LMICs. However, there are important differences that also need to be taken into account when assessing vaccination programmes for LMICs. This guide has outlined many of the influenza vaccine‐specific challenges and should help to provide a framework for future evaluations in the area to build upon.
